# Pseudohyperkalemia and the Need for Imperative Caution With the Newly Introduced Potent Potassium Binders: Two Cases

**DOI:** 10.7759/cureus.17179

**Published:** 2021-08-14

**Authors:** Macaulay A Onuigbo, Adam Ross

**Affiliations:** 1 Internal Medicine, The Robert Larner, M.D. College of Medicine, University of Vermont, Burlington, USA

**Keywords:** erythrocytosis, plasma, pseudohyperkalemia, serum, thrombocytosis, true hyperkalemia

## Abstract

Pseudohyperkalemia was first reported in 1955 by Hartmann and Mellinkoff, as a marked elevation of serum potassium in the absence of clinical evidence of electrolyte imbalance - simultaneous serum potassium exceeds plasma potassium by >0.4 mmol/L. We describe two patients with pseudohyperkalemia who inadvertently received inappropriate potassium binder therapy for weeks to months before the diagnosis of pseudohyperkalemia was entertained and subsequently confirmed. Potassium binders ultimately were promptly discontinued once the diagnosis of pseudohyperkalemia was confirmed. Physicians’ attention must be drawn to the availability of the new potent oral potassium binders, patiromer and sodium zirconium cyclosilicate. We strongly advocate for imperative caution with these new binders. Iatrogenic life-threatening hypokalemia remains a real concern and must be avoided. Our patients highlighted the importance of caution in the use of the newer potent potassium binders to mitigate against the causation of iatrogenic hypokalemia. Also as important is the observation that in the same patient, with changing clinical scenarios, a patient might exhibit true hyperkalemia that alternated with pseudohyperkalemia, the first of such a report.

## Introduction

Pseudohyperkalemia was first reported in 1955 by Hartmann and Mellinkoff as a marked elevation of serum potassium levels in the absence of clinical evidence of electrolyte imbalance [1]. In pseudohyperkalemia, simultaneously measured serum potassium exceeds plasma potassium by >0.4 mmol/L [2]. The impact of specimen handling, transport, and delays in the laboratory testing of blood samples collected from patients is well described in the literature. Pseudohyperkalemia is often associated with moderate to severe thrombocytosis or leukocytosis [3-4]. Unmistakably, true hyperkalemia is potentially lethal. Nevertheless, inappropriate treatment of pseudohyperkalemia leading to severe hypokalemia is also life-threatening [5]. In the first patient, we report a very rare occurrence of pseudohyperkalemia alternating with true hyperkalemia in the same patient during a hospitalization, the first of its kind reported [6]. Recently, new potent and safer potassium binders have been introduced for the management of hyperkalemia [7]. We herein report the utilization of the new potent potassium binders, sodium zirconium cyclosilicate, and patiromer for nearly two weeks and three months, respectively, for the treatment of hyperkalemia before the possibility of pseudohyperkalemia was entertained and subsequently confirmed. Both potassium binders were then promptly discontinued thereafter. We subsequently measured plasma potassium levels after the discontinuation of the potassium binders. Therefore, the degree of iatrogenic hypokalemia that we may have induced in both patients during the use of the potassium binders remains unknown.

## Case presentation

Case 1

**Figure 1:**
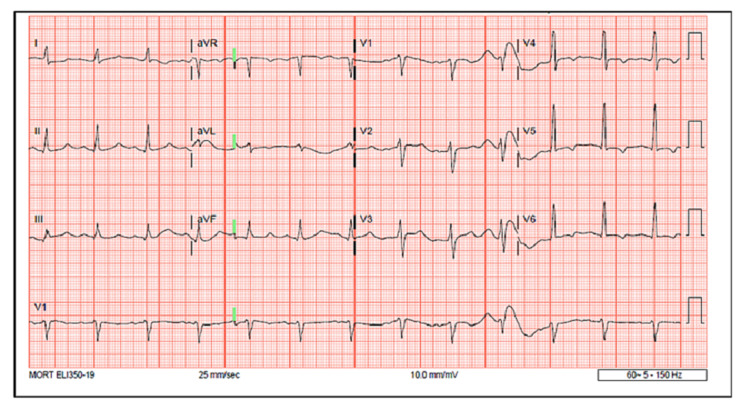
Abnormal EKG tracings in the emergency department in a patient with acute kidney injury, metabolic acidosis, and true hyperkalemia of 7.4 mmol/L showing prolonged QTc duration of 522 (<440) ms and inverted T waves in the lateral leads

We recently described a 40-year-old African American male patient, with sickle cell disease (SS genotype) and sickle cell nephropathy, who was admitted with a painful hemolytic crisis [6]. Electrocardiogram (EKG) in the emergency department revealed a prolonged QTc duration of 522 (<440) ms, inverted T waves in the lateral leads consistent with true hyperkalemia (Figure 1). Emergent measures for hyperkalemia were initiated in the ED and three weeks into the admission, with the acute kidney injury (AKI) resolved, hyperkalemia that had improved from admission had reappeared [6]. Despite daily sodium zirconium cyclosilicate (SZC), chlorthalidone diuretic, and a low potassium diet, hyperkalemia persisted. In the third week of hospitalization, serum potassium of 6.7 mmol/L was recorded, and once again, emergent measures for the treatment of hyperkalemia were reinstituted [6]. Nevertheless, an EKG obtained that morning was normal. This observation triggered the consideration of the diagnosis of pseudohyperkalemia [6]. Pseudohyperkalemia was confirmed when the simultaneously measured serum potassium was 5.8 mmol/L, whereas plasma potassium was 5.2 mmol/L. Therefore, SZC was promptly discontinued and subsequent testing for potassium was by plasma measurements only. He was soon discharged home without any further complications [6].

Figure 1 shows abnormal EKG tracings in the ED during the first hospitalization with AKI, metabolic acidosis, and true hyperkalemia of 7.4 mmol/L.

Case 2

An otherwise active 77-year-old female with controlled hypertension on amlodipine 2.5 mg daily was evaluated by nephrology for hyperkalemia of 5.7 (3.5 - 5.0) mmol/L. Serum creatinine was normal at 0.92 mg/dL. Elevated serum potassium was recorded by her primary physician about five months earlier and had persisted despite very strict dietary restrictions. She monitored her oral potassium intake with the aid of a Smartphone App to 1500 mg/day. She developed pruritus with furosemide, which was prescribed for hyperkalemia, and furosemide was discontinued. She had also complained of intermittent palpitations, fast heart rate, and numbness of the arms and the right leg. She saw a cardiologist and an EKG was non-diagnostic. The historical review revealed that the majority of serum potassium results in the previous five years were elevated (5.1 - 6.2 mEq/L). Prior to April 2015, potassium levels were normal. Blood pressure was normal, and physical examination was unremarkable. She was switched to ethacrynic acid, a non-sulfonamide loop diuretic, 50 mg daily, for hyperkalemia. Morning serum aldosterone, 24-hr urine aldosterone, and plasma renin activity were normal. Until then, the working diagnosis was hyperkalemia from impaired renal potassium excretion, some form of renal tubular acidosis. With persisting hyperkalemia, ethacrynic acid was increased to 50 mg twice daily, but she soon developed nausea, bloating sensation, and excruciating upper abdominal pain. Ethacrynic acid was discontinued with the resolution of these new symptoms. Simultaneously, while on the higher dose of ethacrynic acid, serum creatinine had increased to 1.25 mg/dL and serum potassium had decreased to 4.3 mEq/L. A week following the discontinuation of ethacrynic acid, serum potassium was back up to 5.6 Eq/L despite continued dietary potassium restrictions. Therefore, patiromer, 8.4 g daily, was started about three months after the initial nephrology consultation. She felt better on the patiromer, and her serum potassium level was about 5 mmol/L on rechecks. Three months later, while still on daily patiromer, she presented to the local emergency department with new symptoms of a numb tongue and swelling of the left leg below the knee. Doppler evaluation ruled out left lower extremity deep vein thrombosis (DVT). However, CBC revealed hemoglobin 19.7 g/dL, hematocrit 62.1%, platelets 631 (141-377) k/cmm, and WBC 13.04 k/cmm (80.4% neutrophils). The hepatic function panel was abnormal with a total bilirubin of 1.9 mg/dL, unconjugated bilirubin 1.3 mg/dL, lactate dehydrogenase (LDH) 1,300 (313-618) U/L, with normal albumin, transaminases, and alkaline phosphatase. C-reactive protein (CRP) was <7 mg/L. Notably, the only previous hematology test on record was from February 2001, and the hematocrit then was 39.9%. She was evaluated by hematology. Subsequent peripheral blood testing by quantitative allele-specific polymerase chain reaction (PCR) assay was positive and JAK2 V617F mutated DNA was detected and measured at 62% of total JAK2 DNA. She was finally diagnosed with polycythemia vera (PCV). Treatment included serial phlebotomy procedures followed by hydroxyurea. Two months later, WBC was 10.53 k/cmm, hemoglobin 16.5 (11.6-15.2) g/dL, hematocrit 52.8 (34.9-44.4%), and platelet count 486 k/cmm. Following the diagnosis of PCV, the possibility of pseudohyperkalemia was therefore immediately entertained. Simultaneous serum and plasma potassium concentrations were obtained, while still on patiromer. Serum potassium was 5.2 mmol/L, whereas plasma potassium was only 3.9 mmol/L, thus confirming the diagnosis of pseudohyperkalemia. Patiromer was promptly discontinued. Her low potassium diet was liberalized, and plasma potassium levels have remained normal since then. In the interim, only plasma (and not serum) potassium levels have been continuously monitored while the PCV is being managed by hematology.

## Discussion

We have described two patients with pseudohyperkalemia who inadvertently received inappropriate potassium binder therapy for weeks to months before the diagnosis of pseudohyperkalemia was entertained and subsequently confirmed. The first patient was very peculiar because he had exhibited alternating true hyperkalemia earlier on admission with acute kidney injury and metabolic acidosis complicating acute sickle cell crisis, but later during the same admission, with the recovery of kidney failure and secondary thrombocytosis, he then demonstrated pseudohyperkalemia that was confirmed by differential plasma and serum potassium testing [6]. However, before pseudohyperkalemia was diagnosed, we had treated the patient in the hospital with over one week of the new potassium binder, sodium zirconium cyclosilicate, with monitoring of only serum potassium at the time [6]. The dangers of erroneously treating pseudohyperkalemia include an inappropriate decrease in actual serum potassium levels, which may lead to arrhythmias and other adverse cardiovascular complications [8-11]. Anti-hyperkalemic treatment of patients without true hyperkalemia can produce iatrogenic hypokalemia, which can be fatal [9]. A healthy ECG in the proper clinical setting can assist in making the diagnosis at the bedside [6,11-12]. On the other hand, the second patient must have had undiagnosed polycythemia vera for a long time and such cases of pseudohyperkalemia due to diagnosed and undiagnosed myeloproliferative have been variously reported in the literature [13-18]. Following the approval of patiromer and sodium zirconium cyclosilicate, there is now an increased uptake in the prescribing of these agents [7,19]. The second patient was diagnosed with hyperkalemia and suspected renal tubular acidosis and was treated with patiromer, for several months, again with monitoring of only serum potassium, before the correct diagnosis of pseudohyperkalemia was entertained and confirmed after the diagnosis of polycythemia vera (PCV) was made. In both instances, immediately following the diagnosis of pseudohyperkalemia, the potassium binders were then promptly discontinued. Subsequent testing for potassium was then continued with plasma potassium measurements only. Since we subsequently measured plasma potassium levels after the discontinuation of the potassium binders in both patients, the degree of iatrogenic hypokalemia that we may have induced during the use of the potassium binders remains largely unknown.

In a recently published series from Israel, pseudohyperkalemia was documented in medical charts only in a minority of cases (n = 4, 8.1%) [12]. Treatment was administered in 17 of 49 (34.7%) cases and caused significant hypokalemia in six of those cases [12]. It is imperative to all physicians and other healthcare providers that such iatrogenic and potentially life-threatening hypokalemia must be avoided at all costs.

In the multinational safety trials for patiromer in the OPAL-HK Clinical Trial, the incidence of hypokalemia (serum potassium level <3.5 mmol per liter) was 3.0% [20]. In the phase 3 AMETHYST-DN randomized clinical trial with patiromer in patients with hyperkalemia and diabetic kidney disease, hypokalemia (<3.5 mEq/L) occurred in 5.6% of patients [21]. Similarly, in the phase 3 HARMONIZE randomized clinical trial of sodium zirconium cyclosilicate on potassium lowering for 28 days among outpatients with hyperkalemia, hypokalemia developed in 5/51 (10%) and 6/56 patients (11%) in the 10-g and 15-g zirconium cyclosilicate groups vs none in the 5-g or placebo groups [22]. Clearly, there would be a much higher incidence of potentially life-threatening hypokalemia if and when these new potent potassium binders, patiromer and sodium zirconium cyclosilicate, are used for prolonged periods of time in patients with pseudohyperkalemia [6]. Clearly, with the introduction of these new, potent, and safe potassium binders, they are now being prescribed for prolonged periods of time to enable the continued use of RAAS blockers in medicine [7,19-22]. Before their introduction, sodium polystyrene stearate was only used sparingly, and then for only very short periods of time. Iatrogenic life-threatening hypokalemia, therefore, remains a real concern and must be avoided in patients with pseudohyperkalemia who receive prolonged treatment with the new potent potassium binders, patiromer and sodium zirconium cyclosilicate [7,19].

## Conclusions

Physicians must always entertain the plausibility of pseudohyperkalemia under appropriate clinical scenarios. The first patient had developed thrombocytosis post-sickle cell crisis with pseudohyperkalemia resulting from the thrombocytosis. This was the first time, it was reported that pseudohyperkalemia manifested in the same patient during an index hospitalization following initial true hyperkalemia. As evident from our case reports, one well-tested mantra for all physicians and healthcare providers to always remember is that true hyperkalemia in the absence of overt kidney dysfunction or during recovery from kidney failure must always be viewed with significant circumspection and scrutiny. Such critical scrutiny would have led to earlier consideration of the diagnosis of pseudohyperkalemia in both our case reports. In the first patient, during the third week of the hospitalization, kidney function had normalized. Therefore, the new manifestation of hyperkalemia at this time, with resolved acute kidney injury and resolved metabolic acidosis should have triggered an earlier search for pseudohyperkalemia. The second patient with normal kidney function at the time of presentation should similarly have led to at least some basic hematologic evaluation that would have included a complete blood count. This would have led to the diagnosis of polycythemia vera in the first weeks of the nephrology consultation and would have obviated the prolonged administration of unnecessary diuretics and later months of patiromer. We therefore strongly advocate for imperative caution with these newly introduced oral potassium binders in a bid to avoid potentially lethal iatrogenic hypokalemia with their inappropriate and prolonged use in patients with pseudohyperkalemia.
